# Autonomous chemical research with large language models

**DOI:** 10.1038/s41586-023-06792-0

**Published:** 2023-12-20

**Authors:** Daniil A. Boiko, Robert MacKnight, Ben Kline, Gabe Gomes

**Affiliations:** 1 Department of Chemical Engineering, Carnegie Mellon University, Pittsburgh, PA USA; 2 Emerald Cloud Lab, South San Francisco, CA USA; 3 Department of Chemistry, Carnegie Mellon University, Pittsburgh, PA USA; 4 Wilton E. Scott Institute for Energy Innovation, Carnegie Mellon University, Pittsburgh, PA USA

**Keywords:** Chemistry, Computer science

## Abstract

Transformer-based large language models are making significant strides in various fields, such as natural language processing^1–5^, biology^6,7^, chemistry^8–10^ and computer programming^11,12^. Here, we show the development and capabilities of Coscientist, an artificial intelligence system driven by GPT-4 that autonomously designs, plans and performs complex experiments by incorporating large language models empowered by tools such as internet and documentation search, code execution and experimental automation. Coscientist showcases its potential for accelerating research across six diverse tasks, including the successful reaction optimization of palladium-catalysed cross-couplings, while exhibiting advanced capabilities for (semi-)autonomous experimental design and execution. Our findings demonstrate the versatility, efficacy and explainability of artificial intelligence systems like Coscientist in advancing research.

## Abstract (web summary)

Coscientist is an artificial intelligence system driven by GPT-4 that autonomously designs, plans and performs experiments by incorporating large language models empowered by tools such as internet and documentation search, code execution and experimental automation.

## Main

Large language models (LLMs), particularly transformer-based models, are experiencing rapid advancements in recent years. These models have been successfully applied to various domains, including natural language^1–5^, biological^6,7^ and chemical research^8–10^ as well as code generation^11,12^. Extreme scaling of models^13^, as demonstrated by OpenAI, has led to significant breakthroughs in the field^1,14^. Moreover, techniques such as reinforcement learning from human feedback^15^ can considerably enhance the quality of generated text and the models’ capability to perform diverse tasks while reasoning about their decisions^16^.

On 14 March 2023, OpenAI released their most capable LLM to date, GPT-4^14^. Although specific details about the model training, sizes and data used are limited in GPT-4’s technical report, OpenAI researchers have provided substantial evidence of the model’s exceptional problem-solving abilities. Those include—but are not limited to—high percentiles on the SAT and BAR examinations, LeetCode challenges and contextual explanations from images, including niche jokes^14^. Moreover, the technical report provides an example of how the model can be used to address chemistry-related problems.

Simultaneously, substantial progress has been made toward the automation of chemical research. Examples range from the autonomous discovery^17,18^ and optimization of organic reactions^19^ to the development of automated flow systems^20,21^ and mobile platforms^22^.

The combination of laboratory automation technologies with powerful LLMs opens the door to the development of a sought-after system that autonomously designs and executes scientific experiments. To accomplish this, we intended to address the following questions. What are the capabilities of LLMs in the scientific process? What degree of autonomy can we achieve? How can we understand the decisions made by autonomous agents?

In this work, we present a multi-LLMs-based intelligent agent (hereafter simply called Coscientist) capable of autonomous design, planning and performance of complex scientific experiments. Coscientist can use tools to browse the internet and relevant documentation, use robotic experimentation application programming interfaces (APIs) and leverage other LLMs for various tasks. This work has been done independently and in parallel to other works on autonomous agents^23–25^, with ChemCrow^26^ serving as another example in the chemistry domain. In this paper, we demonstrate the versatility and performance of Coscientist in six tasks: (1) planning chemical syntheses of known compounds using publicly available data; (2) efficiently searching and navigating through extensive hardware documentation; (3) using documentation to execute high-level commands in a cloud laboratory; (4) precisely controlling liquid handling instruments with low-level instructions; (5) tackling complex scientific tasks that demand simultaneous use of multiple hardware modules and integration of diverse data sources; and (6) solving optimization problems requiring analyses of previously collected experimental data.

## Coscientist system architecture

**Fig. 1:**
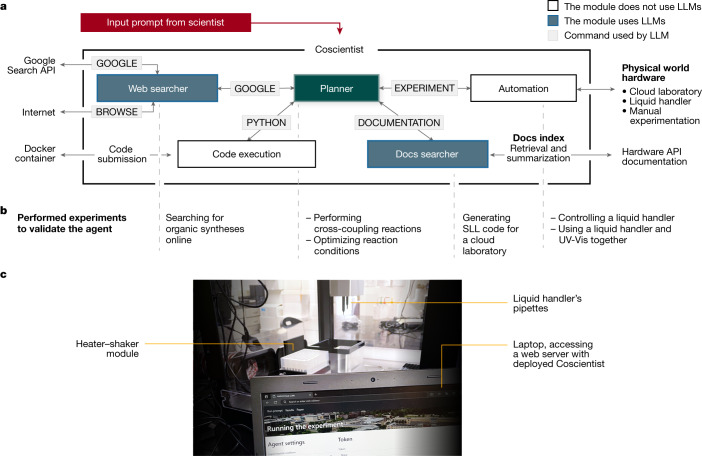
The system’s architecture. **a**, Coscientist is composed of multiple modules that exchange messages. Boxes with blue background represent LLM modules, the Planner module is shown in green, and the input prompt is in red. White boxes represent modules that do not use LLMs. **b**, Types of experiments performed to demonstrate the capabilities when using individual modules or their combinations. **c**, Image of the experimental setup with a liquid handler. UV-Vis, ultraviolet visible.

Coscientist acquires the necessary knowledge to solve a complex problem by interacting with multiple modules (web and documentation search, code execution) and by performing experiments. The main module (‘Planner’) has the goal of planning, based on the user input by invoking the commands defined below. The Planner is a GPT-4 chat completion instance serving the role of an assistant. The initial user input along with command outputs are treated as user messages to the Planner. System prompts (static inputs defining the LLMs’ goals) for the Planner are engineered^1,27^ in a modular fashion, described as four commands that define the action space: ‘GOOGLE’, ‘PYTHON’, ‘DOCUMENTATION’ and ‘EXPERIMENT’. The Planner calls on each of these commands as needed to collect knowledge. The GOOGLE command is responsible for searching the internet with the ‘Web searcher’ module, which is another LLM itself. The PYTHON command allows the Planner to perform calculations to prepare the experiment using a ‘Code execution’ module. The EXPERIMENT command actualizes ‘Automation’ through APIs described by the DOCUMENTATION module. Like GOOGLE, the DOCUMENTATION command provides information to the main module from a source, in this case documentation concerning the desired API. In this study, we have demonstrated the compatibility with the Opentrons Python API and the Emerald Cloud Lab (ECL) Symbolic Lab Language (SLL). Together, these modules make up Coscientist, which receives a simple plain text input prompt from the user (for example, “perform multiple Suzuki reactions”). This architecture is depicted in Fig. 1.

Furthermore, some of the commands can use subactions. The GOOGLE command is capable of transforming prompts into appropriate web search queries, running them against the Google Search API, browsing web pages and funneling answers back to the Planner. Similarly, the DOCUMENTATION command performs retrieval and summarization of necessary documentation (for example, robotic liquid handler or a cloud laboratory) for Planner to invoke the EXPERIMENT command.

The PYTHON command performs code execution (not reliant upon any language model) using an isolated Docker container to protect the users’ machine from any unexpected actions requested by the Planner. Importantly, the language model behind the Planner enables code to be fixed in case of software errors. The same applies to the EXPERIMENT command of the Automation module, which executes generated code on corresponding hardware or provides the synthetic procedure for manual experimentation.

## Web search module

**Fig. 2:**
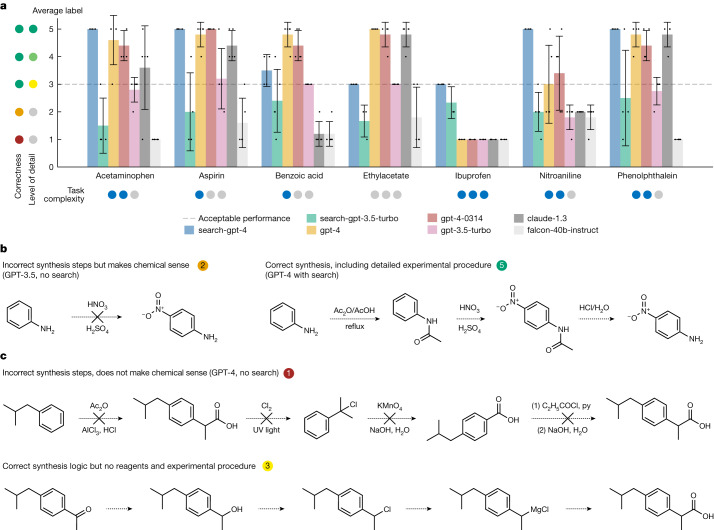
Coscientist’s capabilities in chemical synthesis planning tasks. **a**, Comparison of various LLMs on compound synthesis benchmarks. Error bars represent s.d. values. **b**, Two examples of generated syntheses of nitroaniline. **c**, Two example of generated syntheses of ibuprofen. UV, ultraviolet.

To demonstrate one of the functionalities of the Web Searcher module, we designed a test set composed of seven compounds to synthesize, as presented in Fig. 2a. The Web Searcher module versions are represented as ‘search-gpt-4’ and ‘search-gpt-3.5-turbo’. Our baselines include OpenAI’s GPT-3.5 and GPT-4, Anthropic’s Claude 1.3^28^ and Falcon-40B-Instruct^29^—considered one of the best open-source models at the time of this experiment as per the OpenLLM leaderboard^30^.

We prompted every model to provide a detailed compound synthesis, ranking the outputs on the following scale (Fig. 2):

- 5 for a very detailed and chemically accurate procedure description
- 4 for a detailed and chemically accurate description but without reagent quantities
- 3 for a correct chemistry description that does not include step-by-step procedure
- 2 for extremely vague or unfeasible descriptions
- 1 for incorrect responses or failure to follow instructions

All scores below 3 indicate task failure. It is important to note that all answers between 3 and 5 are chemically correct but offer varying levels of detail. Despite our attempts to better formalize the scale, labelling is inherently subjective and so, may be different between the labelers.

Across non-browsing models, the two versions of the GPT-4 model performed best, with Claude v.1.3 demonstrating similar performance. GPT-3 performed significantly worse, and Falcon 40B failed in most cases. All non-browsing models incorrectly synthesized ibuprofen (Fig. 2c). Nitroaniline is another example; although some generalization of chemical knowledge might inspire the model to propose direct nitration, this approach is not experimentally applicable as it would produce a mixture of compounds with a very minor amount of the product (Fig. 2b). Only the GPT-4 models occasionally provided the correct answer.

The GPT-4-powered Web Searcher significantly improves on synthesis planning. It reached maximum scores across all trials for acetaminophen, aspirin, nitroaniline and phenolphthalein (Fig. 2b). Although it was the only one to achieve the minimum acceptable score of three for ibuprofen, it performed lower than some of the other models for ethylacetate and benzoic acid, possibly because of the widespread nature of these compounds. These results show the importance of grounding LLMs to avoid ‘hallucinations’^31^. Overall, the performance of GPT-3.5-enabled Web Searcher trailed its GPT-4 competition, mainly because of its failure to follow specific instructions regarding output format.

Extending the Planner’s action space to leverage reaction databases, such as Reaxys^32^ or SciFinder^33^, should significantly enhance the system’s performance (especially for multistep syntheses). Alternatively, analysing the system’s previous statements is another approach to improving its accuracy. This can be done through advanced prompting strategies, such as ReAct^34^, Chain of Thought^35^ and Tree of Thoughts^36^.

## Documentation search module

Addressing the complexities of software components and their interactions is crucial for integrating LLMs with laboratory automation. A key challenge lies in enabling Coscientist to effectively utilize technical documentation. LLMs can refine their understanding of common APIs, such as the Opentrons Python API^37^, by interpreting and learning from relevant technical documentation. Furthermore, we show how GPT-4 can learn how to programme in the ECL SLL.

**Fig. 3:**
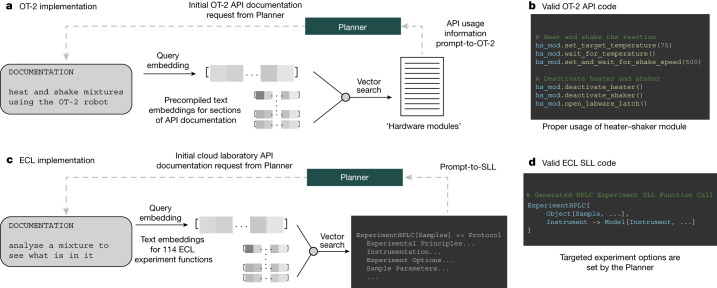
Overview of documentation search. **a**, Prompt-to-code through ada embedding and distance-based vector search. **b**, Example of code for using OT-2’s heater–shaker module. **c**, Prompt-to-function/prompt-to-SLL (to symbolic laboratory language) through supplementation of documentation. **d**, Example of valid ECL SLL code for performing high-performance liquid chromatography (HPLC) experiments.

Our approach involved equipping Coscientist with essential documentation tailored to specific tasks (as illustrated in Fig. 3a), allowing it to refine its accuracy in using the API and improve its performance in automating experiments.

Information retrieval systems are usually based on two candidate selection approaches: inverted search index and vector database^38–41^. For the first one, each unique word in the search index is mapped to the documents containing it. At inference time, all documents containing words from a query are selected and ranked based on various manually defined formulas^42^. The second approach starts by embedding the documents with neural networks or as term frequency–inverse document frequency embedding vectors^43^, followed by the construction of a vector database. Retrieval of similar vectors from this database occurs at inference time, usually using one of the approximate nearest neighbour search algorithms^44^. When strategies such as Transformer models are used, there are more chances to account for synonyms natively without doing synonym-based query expansion, as would be done in the first approach^45^.

Following the second approach, all sections of the OT-2 API documentation were embedded using OpenAI’s ada model. To ensure proper use of the API, an ada embedding for the Planner’s query was generated, and documentation sections are selected through a distance-based vector search. This approach proved critical for providing Coscientist with information about the heater–shaker hardware module necessary for performing chemical reactions (Fig. 3b).

A greater challenge emerges when applying this approach to a more diverse robotic ecosystem, such as the ECL. Nonetheless, we can explore the effectiveness of providing information about the ECL SLL, which is currently unknown to the GPT-4 model. We conducted three separate investigations concerning the SLL: (1) prompt-to-function; (2) prompt-to-SLL; and (3) prompt-to-samples. Those investigations are detailed in Supplementary Information section ‘ECL experiments’.

For investigation 1, we provide the Docs searcher with a documentation guide from ECL pertaining to all available functions for running experiments^46^. Figure 3c summarizes an example of the user providing a simple prompt to the system, with the Planner receiving relevant ECL functions. In all cases, functions are correctly identified for the task.

Figure 3c,d continues to describe investigation 2, the prompt-to-SLL investigation. A single appropriate function is selected for the task, and the documentation is passed through a separate GPT-4 model to perform code retention and summarization. After the complete documentation has been processed, the Planner receives usage information to provide EXPERIMENT code in the SLL. For instance, we provide a simple example that requires the ‘ExperimentHPLC’ function. Proper use of this function requires familiarity with specific ‘Models’ and ‘Objects’ as they are defined in the SLL. Generated code was successfully executed at ECL; this is available in Supplementary Information. The sample was a caffeine standard sample. Other parameters (column, mobile phases, gradients) were determined by ECL’s internal software (a high-level description is in Supplementary Information section ‘HPLC experiment parameter estimation’). Results of the experiment are provided in Supplementary Information section ‘Results of the HPLC experiment in the cloud lab’. One can see that the air bubble was injected along with the analyte’s solution. This demonstrates the importance of development of automated techniques for quality control in cloud laboratories. Follow-up experiments leveraging web search to specify and/or refine additional experimental parameters (column chemistry, buffer system, gradient and so on) would be required to optimize the experimental results. Further details on this investigation are in Supplementary Information section ‘Analysis of ECL documentation search results’.

A separate prompt-to-samples investigation, investigation 3, was conducted by providing a catalogue of available samples, enabling the identification of relevant stock solutions that are on ECL’s shelves. To showcase this feature, we provide the Docs searcher module with all 1,110 Model samples from the catalogue. By simply providing a search term (for example, ‘Acetonitrile’), all relevant samples are returned. This is also available in Supplementary Information.

## Controlling laboratory hardware

**Fig. 4:**
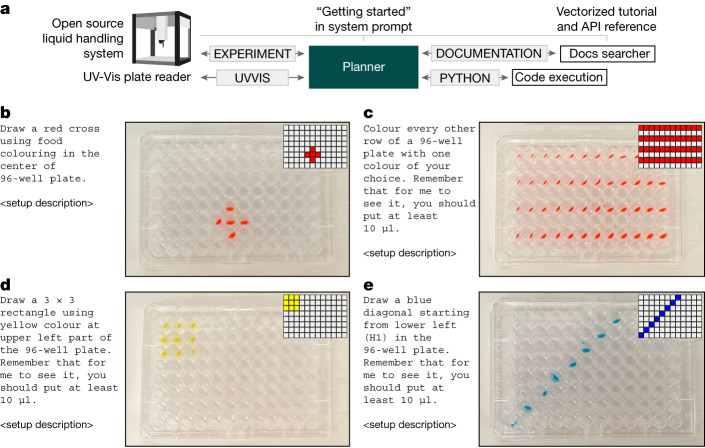
Robotic liquid handler control capabilities and integration with analytical tools. **a**, Overview of Coscientist’s configuration. **b**, Drawing a red cross. **c**, Colouring every other row. **d**, Drawing a yellow rectangle. **e**, Drawing a blue diagonal.

Access to documentation enables us to provide sufficient information for Coscientist to conduct experiments in the physical world. To initiate the investigation, we chose the Opentrons OT-2, an open-source liquid handler with a well-documented Python API. The ‘Getting Started’ page from its documentation was supplied to the Planner in the system prompt. Other pages were vectorized using the approach described above. For this investigation, we did not grant access to the internet (Fig. 4a).

We started with simple plate layout-specific experiments. Straightforward prompts in natural language, such as “colour every other line with one colour of your choice”, resulted in accurate protocols. When executed by the robot, these protocols closely resembled the requested prompt (Fig. 4b–e).

Ultimately, we aimed to assess the system’s ability to integrate multiple modules simultaneously. Specifically, we provided the ‘UVVIS’ command, which can be used to pass a microplate to plate reader working in the ultraviolet–visible wavelength range. To evaluate Coscientist’s capabilities to use multiple hardware tools, we designed a toy task; in 3 wells of a 96-well plate, three different colours are present—red, yellow and blue. The system must determine the colours and their positions on the plate without any prior information.

**Extended Data Fig. 1:**
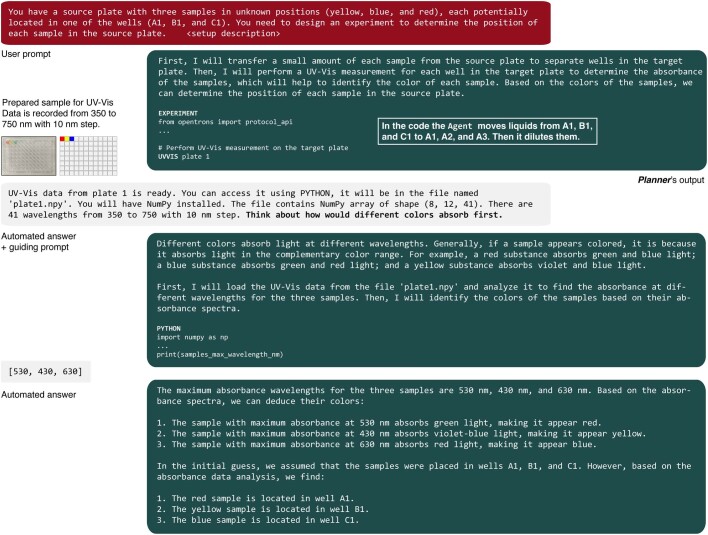
Using UV-Vis and liquid handler to solve food colouring identification problem. Guiding prompt in the third message is shown in bold. In the first message the user prompt is provided, then code for sample preparation is generated, resulting data is provided as NumPy array, which is then analysed to give the final answer.

The Coscientist’s first action was to prepare small samples of the original solutions (Extended Data Fig. 1). Ultraviolet-visible measurements were then requested to be performed by the Coscientist (Supplementary Information section ‘Solving the colours problem’ and Supplementary Fig. 1). Once completed, Coscientist was provided with a file name containing a NumPy array with spectra for each well of the microplate. Coscientist subsequently generated Python code to identify the wavelengths with maximum absorbance and used these data to correctly solve the problem, although it required a guiding prompt asking it to think through how different colours absorb light.

## Integrated chemical experiment design

**Fig. 5:**
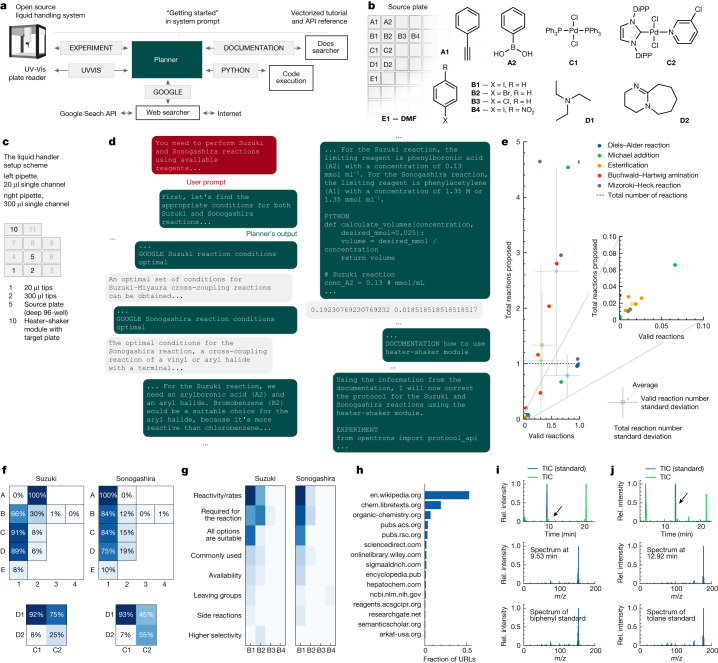
Cross-coupling Suzuki and Sonogashira reaction experiments designed and performed by Coscientist. **a**, Overview of Coscientist’s configuration. **b**, Available compounds (DMF, dimethylformamide; DiPP, 2,6-diisopropylphenyl). **c**, Liquid handler setup. **d**, Solving the synthesis problem. **e**, Comparison of reagent selection performance with a large dataset. **f**, Comparison of reagent choices across multiple runs. **g**, Overview of justifications made when selecting various aryl halides. **h**, Frequency of visited URLs. **i**, Total ion current (TIC) chromatogram of the Suzuki reaction mixture (top panel) and the pure standard, mass spectra at 9.53 min (middle panel) representing the expected reaction product and mass spectra of the pure standard (bottom panel). **j**, TIC chromatogram of the Sonogashira reaction mixture (top panel) and the pure standard, mass spectra at 12.92 min (middle panel) representing the expected reaction product and mass spectra of the pure standard (bottom panel). Rel., relative.

We evaluated Coscientist’s ability to plan catalytic cross-coupling experiments by using data from the internet, performing the necessary calculations and ultimately, writing code for the liquid handler. To increase complexity, we asked Coscientist to use the OT-2 heater–shaker module released after the GPT-4 training data collection cutoff. The available commands and actions supplied to the Coscientist are shown in Fig. 5a. Although our setup is not yet fully automated (plates were moved manually), no human decision-making was involved.

The test challenge for Coscientist’s complex chemical experimentation capabilities was designed as follows. (1) Coscientist is provided with a liquid handler equipped with two microplates (source and target plates). (2) The source plate contains stock solutions of multiple reagents, including phenyl acetylene and phenylboronic acid, multiple aryl halide coupling partners, two catalysts, two bases and the solvent to dissolve the sample (Fig. 5b). (3) The target plate is installed on the OT-2 heater–shaker module (Fig. 5c). (4) Coscientist’s goal is to successfully design and perform a protocol for Suzuki–Miyaura and Sonogashira coupling reactions given the available resources.

To start, Coscientist searches the internet for information on the requested reactions, their stoichiometries and conditions (Fig. 5d). The correct coupling partners are selected for the corresponding reactions. Designing and performing the requested experiments, the strategy of Coscientist changes among runs (Fig. 5f). Importantly, the system does not make chemistry mistakes (for instance, it never selects phenylboronic acid for the Sonogashira reaction). Interestingly, the base DBU (1,8-diazabicyclo[5.4.0]undec-7-ene) is selected more often with the PEPPSI–IPr (PEPPSI, pyridine-enhanced precatalyst preparation stabilization and initiation; IPr, 1,3-bis(2,6-diisopropylphenyl)imidazol-2-ylidene) complex, with that preference switching in Sonogashira reaction experiments; likewise, bromobenzene is chosen more often for Suzuki than for Sonogashira couplings. Additionally, the model can provide justifications on specific choices (Fig. 5g), demonstrating the ability to operate with concepts such as reactivity and selectivity (more details are in Supplementary Information section ‘Analysis of behaviour across multiple runs’). This capability highlights a potential future use case to analyse the reasoning of the LLMs used by performing experiments multiple times. Although the Web Searcher visited various websites (Fig. 5h), overall Coscientist retrieves Wikipedia pages in approximately half of cases; notably, American Chemical Society and Royal Society of Chemistry journals are amongst the top five sources.

**Extended Data Fig. 2:**
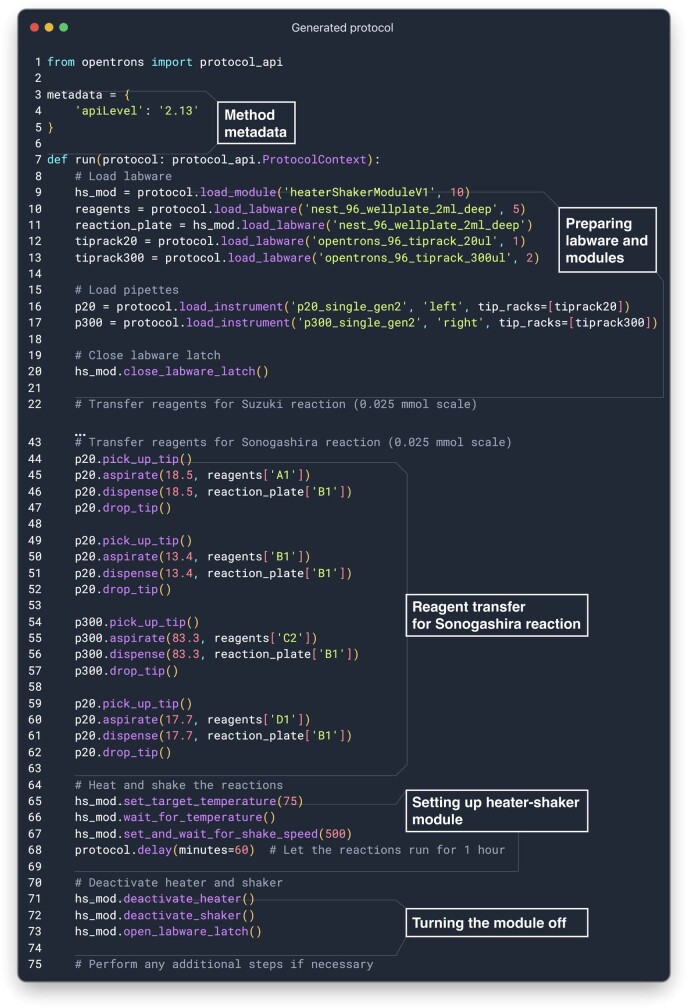
Code, generated by Coscientist. The generated code can be split into the following steps: defining metadata for the method, loading labware modules, setting up the liquid handler, performing required reagent transfers, setting up the heater-shaker module, running the reaction, and turning the module off.

Coscientist then calculates the required volumes of all reactants and writes a Python protocol for running the experiment on the OT-2 robot. However, an incorrect heater–shaker module method name was used. Upon making this mistake, Coscientist uses the Docs searcher module to consult the OT-2 documentation. Next, Coscientist modifies the protocol to a corrected version, which ran successfully (Extended Data Fig. 2). Subsequent gas chromatography–mass spectrometry analysis of the reaction mixtures revealed the formation of the target products for both reactions. For the Suzuki reaction, there is a signal in the chromatogram at 9.53 min where the mass spectra match the mass spectra for biphenyl (corresponding molecular ion mass-to-charge ratio and fragment at 76 Da) (Fig. 5i). For the Sonogashira reaction, we see a signal at 12.92 min with a matching molecular ion mass-to-charge ratio; the fragmentation pattern also looks very close to the one from the spectra of the reference compound (Fig. 5j). Details are in Supplementary Information section ‘Results of the experimental study’.

Although this example requires Coscientist to reason on which reagents are most suitable, our experimental capabilities at that point limited the possible compound space to be explored. To address this, we performed several computational experiments to evaluate how a similar approach can be used to retrieve compounds from large compound libraries^47^. Figure 5e shows Coscientist’s performance across five common organic transformations, with outcomes depending on the queried reaction and its specific run (the GitHub repository has more details). For each reaction, Coscientist was tasked with generating reactions for compounds from a simplified molecular-input line-entry system (SMILES) database. To achieve the task, Coscientist uses web search and code execution with the RDKit chemoinformatics package.

## Chemical reasoning capabilities

The system demonstrates appreciable reasoning capabilities, enabling the request of necessary information, solving of multistep problems and generation of code for experimental design. Some researchers believe that the community is only starting to understand all the capabilities of GPT-4 (ref. ^48^). OpenAI has shown that GPT-4 could rely on some of those capabilities to take actions in the physical world during their initial red team testing performed by the Alignment Research Center^14^.

One of the possible strategies to evaluate an intelligent agent’s reasoning capabilities is to test if it can use previously collected data to guide future actions. Here, we focused on the multi-variable design and optimization of Pd-catalysed transformations, showcasing Coscientist’s abilities to tackle real-world experimental campaigns involving thousands of examples. Instead of connecting LLMs to an optimization algorithm as previously done by Ramos et al.^49^, we aimed to use Coscientist directly.

**Fig. 6:**
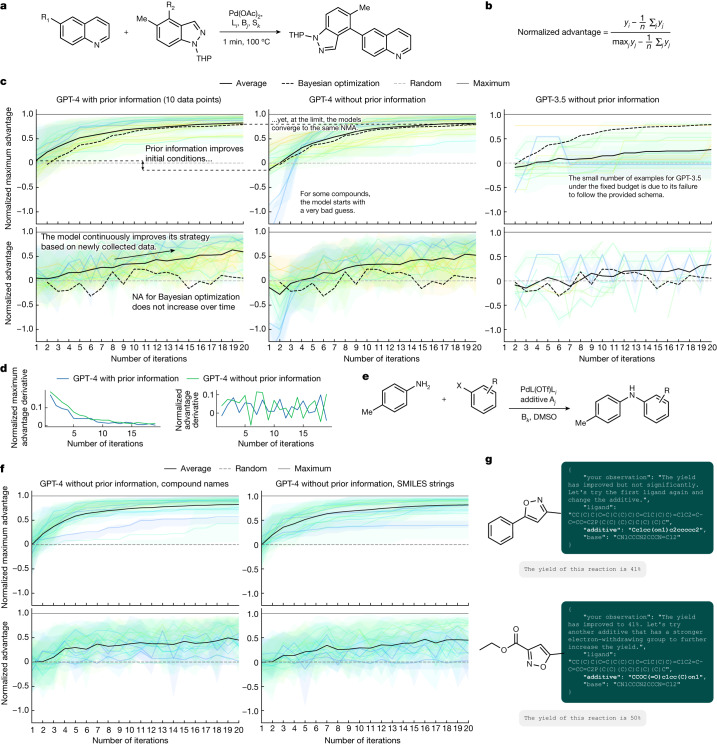
Results of the optimization experiments. **a**, A general reaction scheme from the flow synthesis dataset analysed in **c** and **d**. **b**, The mathematical expression used to calculate normalized advantage values. **c**, Comparison of the three approaches (GPT-4 with prior information, GPT-4 without prior information and GPT-3.5 without prior information) used to perform the optimization process. **d**, Derivatives of the NMA and normalized advantage values evaluated in **c**, left and centre panels. **e**, Reaction from the C–N cross-coupling dataset analysed in **f** and **g**. **f**, Comparison of two approaches using compound names and SMILES string as compound representations. **g**, Coscientist can reason about electronic properties of the compounds, even when those are represented as SMILES strings. DMSO, dimethyl sulfoxide.

We selected two datasets containing fully mapped reaction condition spaces where yield was available for all combinations of variables. One is a Suzuki reaction dataset collected by Perera et al.^50^, where these reactions were performed in flow with varying ligands, reagents/bases and solvents (Fig. 6a). Another is Doyle’s Buchwald–Hartwig reaction dataset^51^ (Fig. 6e), where variations in ligands, additives and bases were recorded. At this point, any reaction proposed by Coscientist would be within these datasets and accessible as a lookup table.

We designed the Coscientist’s chemical reasoning capabilities test as a game with the goal of maximizing the reaction yield. The game’s actions consisted of selecting specific reaction conditions with a sensible chemical explanation while listing the player’s observations about the outcome of the previous iteration. The only hard rule was for the player to provide its actions written in JavaScript Object Notation (JSON) format. If the JSON file could not be parsed, the player is alerted of its failure to follow the specified data format. The player had a maximum of 20 iterations (accounting for 5.2% and 6.9% of the total space for the first and second datasets, respectively) to finish the game.

We evaluate Coscientist’s performance using the normalized advantage metric (Fig. 6b). Advantage is defined as the difference between a given iteration yield and the average yield (advantage over a random strategy). Normalized advantage measures the ratio between advantage and maximum advantage (that is, the difference between the maximum and average yield). The normalized advantage metric has a value of one if the maximum yield is reached, zero if the system exhibits completely random behaviour and less than zero if the performance at this step is worse than random. An increase in normalized advantage over each iteration demonstrates Coscientist’s chemical reasoning capabilities. The best result for a given iteration can be evaluated using the normalized maximum advantage (NMA), which is the normalized value of the maximum advantage achieved until the current step. As NMA cannot decrease, the valuable observations come in the form of the rate of its increase and its final point. Finally, during the first step, the values for NMA and normalized advantage equal each other, portraying the model’s prior knowledge (or lack thereof) without any data being collected.

For the Suzuki dataset, we compared three separate approaches: (1) GPT-4 with prior information included in the prompt (which consisted of 10 yields from random combinations of reagents); (2) GPT-4; or (3) GPT-3.5 without any prior information (Fig. 6c). When comparing GPT-4 with the inclusion and exclusion of prior information, it is clear that the initial guess for the former scenario is better, which aligns with our expectations considering the provided information about the system’s reactivity. Notably, when excluding prior information, there are some poor initial guesses, whereas there are none when the model has prior information. However, at the limit, the models converge to the same NMA. The GPT-3.5 model plots have a very limited number of data points, primarily because of its inability to output messages in the correct JSON schema as requested in the prompt. It is unclear if the GPT-4 training data contain any information from these datasets. If so, one would expect that the initial model guess would be better than what we observed.

The normalized advantage values increase over time, suggesting that the model can effectively reuse the information obtained to provide more specific guidance on reactivity. Evaluating the derivative plots (Fig. 6d) does not show any significant difference between instances with and without the input of prior information.

**Extended Data Fig. 3:**
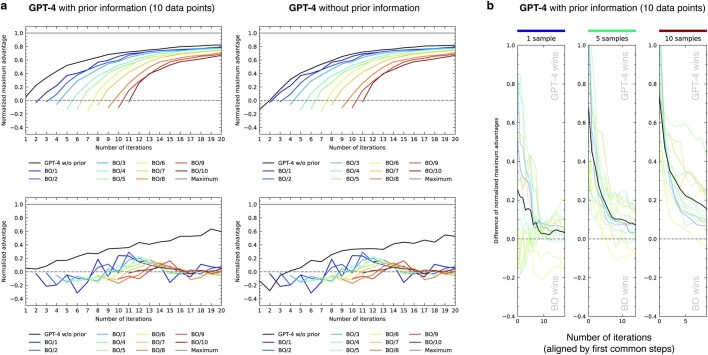
Additional results on comparison with Bayesian optimization. **a**, GPT-4 models compared with Bayesian optimization performed starting with different number of initial samples. **b**, Compound-by-compound comparison of differences between advantages.

There are many established optimization algorithms for chemical reactions. In comparison with standard Bayesian optimization^52^, both GPT-4-based approaches show higher NMA and normalized advantage values (Fig. 6c). A detailed overview of the exact Bayesian optimization strategy used is provided in Supplementary Information section ‘Bayesian optimization procedure’. It is observed that Bayesian optimization’s normalized advantage line stays around zero and does not increase over time. This may be caused by different exploration/exploitation balance for these two approaches and may not be indicative of their performance. For this purpose, the NMA plot should be used. Changing the number of initial samples does not improve the Bayesian optimization trajectory (Extended Data Fig. 3a). Finally, this performance trend is observed for each unique substrate pairings (Extended Data Fig. 3b).

For the Buchwald–Hartwig dataset (Fig. 6e), we compared a version of GPT-4 without prior information operating over compound names or over compound SMILES strings. It is evident that both instances have very similar performance levels (Fig. 6f). However, in certain scenarios, the model demonstrates the ability to reason about the reactivity of these compounds simply by being provided their SMILES strings (Fig. 6g).

## Discussion

In this paper, we presented a proof of concept for an artificial intelligent agent system capable of (semi-)autonomously designing, planning and multistep executing scientific experiments. Our system demonstrates advanced reasoning and experimental design capabilities, addressing complex scientific problems and generating high-quality code. These capabilities emerge when LLMs gain access to relevant research tools, such as internet and documentation search, coding environments and robotic experimentation platforms. The development of more integrated scientific tools for LLMs has potential to greatly accelerate new discoveries.

The development of new intelligent agent systems and automated methods for conducting scientific experiments raises potential concerns about the safety and potential dual-use consequences, particularly in relation to the proliferation of illicit activities and security threats. By ensuring the ethical and responsible use of these powerful tools, we are continuing to explore the vast potential of LLMs in advancing scientific research while mitigating the risks associated with their misuse. A brief dual-use study of Coscientist is provided in Supplementary Information section ‘Safety implications: Dual-use study’.

### Technology use disclosure

The writing of the preprint version of this manuscript was assisted by ChatGPT (specifically, GPT-4 being used for grammar and typos). All authors have read, corrected and verified all information presented in this manuscript and Supplementary Information.

## Online content

Any methods, additional references, Nature Portfolio reporting summaries, source data, extended data, supplementary information, acknowledgements, peer review information; details of author contributions and competing interests; and statements of data and code availability are available at 10.1038/s41586-023-06792-0.

### Supplementary information

Supplementary Information Supplementary Text and Figs. 1–3.

## Data Availability

Examples of the experiments discussed in the text are provided in the Supplementary Information. Because of safety concerns, data, code and prompts will be only fully released after the development of US regulations in the field of artificial intelligence and its scientific applications. Nevertheless, the outcomes of this work can be reproduced using actively developed frameworks for autonomous agent development. The reviewers had access to the web application and were able to verify any statements related to this work. Moreover, we provide a simpler implementation of the described approach, which, although it may not produce the same results, allows for deeper understanding of the strategies used in this work.

## Code availability

Simpler implementation as well as generated outputs used for quantitative analysis are provided at https://github.com/gomesgroup/coscientist.
